# Ultrasound subclinical synovitis in anti-CCP-positive at-risk individuals with musculoskeletal symptoms: an important and predictable stage in the rheumatoid arthritis continuum

**DOI:** 10.1093/rheumatology/keab862

**Published:** 2021-11-24

**Authors:** Andrea Di Matteo, Laurence Duquenne, Edoardo Cipolletta, Jacqueline L Nam, Leticia Garcia-Montoya, Richard J Wakefield, Michael Mahler, Kulveer Mankia, Paul Emery

**Affiliations:** Leeds Institute of Rheumatic and Musculoskeletal Medicine, University of Leeds; National Institute for Health Research, Leeds Biomedical Research Centre, Leeds Teaching Hospitals NHS Trust, Leeds, UK; Rheumatology Unit, Department of Clinical and Molecular Sciences, ‘Carlo Urbani’ Hospital, Polytechnic University of Marche,Jesi, Ancona, Italy; Leeds Institute of Rheumatic and Musculoskeletal Medicine, University of Leeds; National Institute for Health Research, Leeds Biomedical Research Centre, Leeds Teaching Hospitals NHS Trust, Leeds, UK; Rheumatology Unit, Department of Clinical and Molecular Sciences, ‘Carlo Urbani’ Hospital, Polytechnic University of Marche,Jesi, Ancona, Italy; Leeds Institute of Rheumatic and Musculoskeletal Medicine, University of Leeds; National Institute for Health Research, Leeds Biomedical Research Centre, Leeds Teaching Hospitals NHS Trust, Leeds, UK; Leeds Institute of Rheumatic and Musculoskeletal Medicine, University of Leeds; National Institute for Health Research, Leeds Biomedical Research Centre, Leeds Teaching Hospitals NHS Trust, Leeds, UK; Leeds Institute of Rheumatic and Musculoskeletal Medicine, University of Leeds; National Institute for Health Research, Leeds Biomedical Research Centre, Leeds Teaching Hospitals NHS Trust, Leeds, UK; Research and Development, Werfen Autoimmunity, San Diego, CA, USA; Leeds Institute of Rheumatic and Musculoskeletal Medicine, University of Leeds; National Institute for Health Research, Leeds Biomedical Research Centre, Leeds Teaching Hospitals NHS Trust, Leeds, UK; Leeds Institute of Rheumatic and Musculoskeletal Medicine, University of Leeds; National Institute for Health Research, Leeds Biomedical Research Centre, Leeds Teaching Hospitals NHS Trust, Leeds, UK

**Keywords:** ACPA, ultrasound, at-risk, third-generation anti-CCP antibodies, anti-CCP3, subclinical synovitis, prediction, rheumatoid arthritis, inflammatory arthritis

## Abstract

**Objectives:**

To investigate whether anti-CCP2-positive at-risk individuals with musculoskeletal (MSK) symptoms but without clinical synovitis (CCP2^+^ at-risk) develop US subclinical synovitis before inflammatory arthritis and if US subclinical synovitis can be predicted.

**Methods:**

First, US scans of CCP2^+^ at-risk individuals who developed inflammatory arthritis (‘progressors’) were reviewed for subclinical synovitis prior to inflammatory arthritis development. Patients in whom the pre-progression US scan was negative but the scan was conducted >6 months before progression were excluded. Subsequently, regression analyses were performed to identify predictors of US synovitis in CCP2^+^ at-risk individuals without baseline US abnormalities who had one or more longitudinal US scan and a complete dataset.

**Results:**

US subclinical synovitis was detected in one or more scan in 75 of 97 progressors (77.3%) {median time to inflammatory arthritis development from first evidence of US synovitis 26.5 weeks [interquartile range (IQR) 7–60]}, in whom one or more scan was available, excluding those with a negative scan >6 months from inflammatory arthritis development (*n* = 38). In 220 CCP2^+^ at-risk individuals with normal baseline US scans, who had one or more longitudinal US scan and a complete dataset, US synovitis was detected in 69/220 (31.4%) [median time to first developing US synovitis 56.4 weeks (IQR 33.0–112.0)]. In the multivariable analysis, only anti-CCP3 antibodies were predictive for the development of US synovitis [odds ratio 4.75 (95% CI 1.97, 11.46); *P* < 0.01].

**Conclusions:**

In anti-CCP2^+^ at-risk individuals, a stage of subclinical synovitis usually precedes the development of inflammatory arthritis. Anti-CCP2^+^/CCP3^+^ individuals without clinical or US subclinical synovitis may represent the optimal window of opportunity for intervention to prevent joint disease.

Rheumatology key messages

- The majority of anti-CCP2-positive at-risk individuals go through a stage of subclinical synovitis before the development of inflammatory arthritis.
- CCP2-positive at-risk individuals can be identified prior to any joint involvement by the presence of anti-CCP3 antibodies.
- This may represent the optimal ‘window of opportunity’ for intervention to prevent joint disease.

## Introduction

The concept of early RA has recently evolved. It is now considered a ‘disease continuum’ rather than a fixed phenotype, in which individuals with risk factors progress through different stages before the development of clinical arthritis [1–3].

**Fig. 1:**
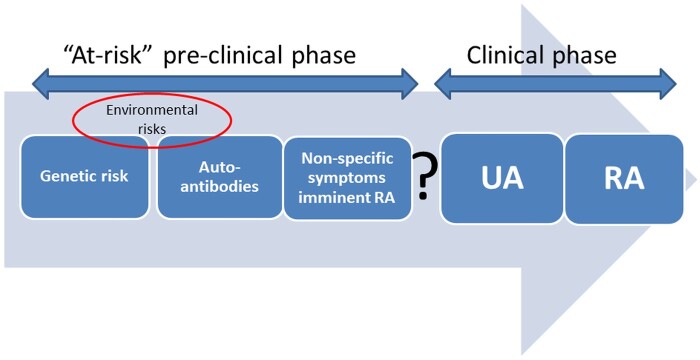
Categories along the RA ‘continuum' defined by the EULAR Standing Committee on Investigative Rheumatology UA: undifferentiated arthritis.

In 2012, the EULAR Standing Committee on Investigative Rheumatology defined six categories along the preclinical ‘RA continuum’: genetic (phase A) and environmental (phase B) risk factors for RA, RA-related systemic autoimmunity (phase C), musculoskeletal (MSK) symptoms without clinical arthritis (phase D), undifferentiated arthritis (phase E) and RA (phase F) [4] (Fig. 1).

Recently, several studies have highlighted the presence of another important population along this ‘continuum', which sits between phases D and E. This is represented by at-risk individuals (i.e. those with systemic autoimmunity and MSK symptoms but without clinical arthritis) who have subclinical joint inflammation on US or MRI [5–7]. These studies have demonstrated that high-resolution imaging is able to detect subclinical joint inflammation in at-risk individuals before clinical synovitis occurs. Indeed, the detection of baseline subclinical joint inflammation and/or joint damage on US has been demonstrated to greatly increase the risk of progression to inflammatory arthritis in at-risk individuals [8–11]. When US power Doppler (PD) is detectable in four or more joints, the future development of RA is almost certain, suggesting this occurs at a late stage in the ‘continuum' [12]. An additional and important aspect that has emerged is that the occurrence of MSK symptoms seems to precede, in most at-risk individuals, the development of subclinical joint inflammation and/or joint damage on US [13, 14]. Symptoms in the absence of clinical or subclinical inflammation may represent the critical time point for preventive treatments, particularly for joint disease.

The identification of those individuals who are at imminent risk, ideally in the window before the occurrence of any joint involvement (i.e. before the ‘second hit’ of RA occurs), is of utmost importance for risk stratification and consequently for disease prevention. However, this is challenging to achieve and therefore biomarkers that can identify those individuals who will develop joint disease would be of great value.

The aims of this study were 2-fold: to investigate whether US subclinical synovitis represents a distinct stage of the ‘continuum’ in second-generation IgG anti-CCP antibody–positive (CCP2^+^) at-risk individuals prior to the development of clinical inflammatory arthritis and to determine in anti-CCP2^+^ at-risk individuals with MSK symptoms but before joint involvement (neither clinical nor subclinical synovitis), clinical and/or serological predictors of US subclinical synovitis.

## Methods

CCP2^+^ (BioPlex 2200, Bio-Rad Laboratories, Hercules, CA, USA) at-risk individuals with MSK symptoms, but without clinical synovitis, taking part in the Leeds CCP study from June 2008 to March 2020 were included. Full details of ‘The CCP Study: Coordinated Programme to Prevent Arthritis – Can We Identify Arthritis at a Pre-clinical Stage?’ have been previously reported [15, 16]. Briefly, in this national study, individuals ≥18 years of age with new MSK symptoms, who test positive for anti-CCP antibodies, are invited to a dedicated research clinic at Chapel Allerton Hospital (Leeds, UK) as part of an observational study. Anti-CCP2 antibody is the most used test to detect ACPA in many places, including the UK. Different from some other at-risk cohorts currently being followed internationally [17, 18], all subjects in the Leeds CCP study are anti-CCP2^+^.

In anti-CCP2^+^ at-risk individuals, the following data were collected at baseline: age, gender, early morning stiffness (EMS) duration, tenderness in the small joints of the hands on physical examination, anti-CCP2 level, third-generation IgG anti-CCP antibody (anti-CCP3) level (QUANTA Lite CCP3, Inova Diagnostic, San Diego, CA, USA) and IgM RF status (BNII nephelometry before February 2010, AdviaXPT turbidometry after February 2010; Siemens, Munich, Germany).

The anti-CCP2 and anti-CCP3 test positivity threshold was set according to the manufacturer’s cut-offs (>2.99 IU/ml and ≥20 units, respectively). The anti-CCP2 level was considered low or high when it was <3 or ≥3 times the positivity threshold, respectively, according to the ACR/EULAR 2010 criteria [19].

A full US protocol was performed as part of the Leeds CCP study. The following joints were scanned: elbows, wrists, first–fifth MCP joints, first–fifth PIP joints, knees, ankles and first–fifth MTP joints. The first MTP joint was not included in the analyses, as US abnormalities have been frequently detected at this level in other non-inflammatory joint diseases, such as OA, as well as in asymptomatic healthy subjects [20, 21]. US synovitis (synovial hypertrophy ≥1 and PD signal ≥1) and bone erosions were identified according to the EULAR/OMERACT and OMERACT definitions, respectively [22, 23]. Three different US machines were used during the study: an ATL HDI 5000 (Philips US, Cambridge, MA, USA), employing 5–12 MHz and 8–15 MHz transducers, and an S7 and Logiq E9 (GE Healthcare, Chicago, IL, USA), both employing a 6–15 MHz transducer. PD was set as follows: pulse repetition frequency (PRF) 700–1000 Hz, Doppler frequency 6 MHz for the ATL HDI 5000 and 10 MHz for the S7 and Logic E9. Sensitivity analyses between the first two US machines (ATL HDI 5000 and S7) were performed due to the change in the US machine during the study [8]. Given the positive results of this analyses and the fact that the S7 was replaced with another GE machine (Logiq E9), the same analyses were not repeated for the third US machine used.

This study consisted of two parts. In the first part (US subclinical synovitis prior to the development of inflammatory arthritis), among at-risk individuals who progressed to inflammatory arthritis, the prevalence and distribution of subclinical synovitis was evaluated in the US scans conducted prior to inflammatory arthritis development. Patients in whom the pre-progression US scan was negative, but the scan was conducted >6 months before progression, were excluded. The reason for this choice was based on the fact that the majority of anti-CCP2^+^ individuals with US synovitis who progress to inflammatory arthritis do so within a short-term follow-up (i.e. median 7.9–9.9 months) [15, 16]. Therefore, individuals with a negative US scan several months before progression may well have subsequently developed an undetected subclinical synovitis. In the second part of the study (predicting the development of US subclinical synovitis), we selected only anti-CCP2^+^ individuals without US abnormalities at baseline (i.e. neither US synovitis nor bone erosions) who had one or more longitudinal US scan and in whom a complete dataset was available. In these patients, we investigated predictors of subclinical synovitis on longitudinal scans.

This study was approved by the NHS Health Research Authority National Research Ethics Service Committee Yorkshire & the Humber–Leeds West. All individuals participating in the study provided full written informed consent.

### Statistical analysis

Descriptive statistics were used to describe the main characteristics of the study population and reported as absolute frequencies with the corresponding percentage for categorical variables, mean (s.d.) for continuous variables with a normal distribution and median with interquartile range (IQR) for non-normally distributed continuous variables. The chi-squared test was used for comparing categorical variables. The Mann–Whitney *U* test was used to compare continuous variables. A univariable analysis was performed to define the predictive value of age, gender, anti-CCP2 antibodies (high/low), anti-CCP3 antibodies (positive/negative), RF (positive/negative), EMS >30 min and tenderness in the small joints of the hands on physical examination for the development of US subclinical synovitis at follow-up. The multivariable regression analysis was adjusted for those parameters that were significant in the univariable analysis. In addition, we performed three different multivariable models excluding either anti-CCP2 (high level), anti-CCP3 or RF, to rule out the potential influence of the collinearity between these variables on the multivariable analysis results. The collinearity between anti-CCP2, anti-CCP3 and RF was also explored using Cramér’s V. A coefficient >0.60 was considered to be indicative of collinearity. Kaplan–Meier analysis and logrank tests were performed to evaluate the US subclinical synovitis-free survival time for anti-CCP3 antibodies and RF. Statistical analyses were performed using SPSS version 25.0 for Windows (IBM, Armonk, NY, USA). The level of significance was set at 5%.

## Results

### US subclinical synovitis prior to the development of inflammatory arthritis

A total of 155/620 (25.0%) anti-CCP2^+^ at-risk individuals progressed to inflammatory arthritis [median time to develop inflammatory arthritis from the baseline visit: 51 weeks (IQR 24–107.2)]. At least one US scan performed prior to the development of clinical arthritis was available in 135/155 progressors (87.1%). Thirty-eight individuals in whom the most recent scan before progression was negative and where this scan was >6 months before progression were excluded. US subclinical synovitis was detected in one or more scan in 75 of the remaining 97 individuals (77.3%) [median time to inflammatory arthritis development from first developing US synovitis: 26.5 weeks (IQR 7.0–60.0); median number of joints with US synovitis: 2.0 (IQR 1.0–3.0)].

The demographic and clinical characteristics of progressors with one or more US scan available is reported in Table 1.

**Table 1:** Baseline demographic and clinical characteristics of the anti-CCP2^+^ at-risk individuals who developed inflammatory arthritis

Characteristics		Total population (*N* = 135)	US subclinical synovitis preceding clinical inflammatory arthritis (*n* = 75)	No US subclinical synovitis preceding clinical inflammatory arthritis (*n* = 22)	Individuals not included in the analysis^*^ (*n* = 38)
Age, years, mean (s.d.)		53.9 (12.8)	56.3 (12.8)	50.0 (10.5)	51.5 (12.4)
Female, *n* (%)		102 (75.5)	54 (72.0)	15 (68.2)	33 (86.8)
Tenderness in the hands, *n* (%)		62 (45.9)	38 (50.7)	8 (36.4)	16 (42.1)
EMS, minutes, median (IQR)		15 (0–60)	15 (0–42.5)	37.5 (15–60)	10 (0–30)
Anti-CCP2 antibodies, *n* (%)	Low	16 (11.9)	11 (14.7)	1 (4.5)	4 (10.5)
	High	119 (88.1)	64 (85.3)	21 (95.5)	34 (89.5)
Anti-CCP3 antibodies, *n* (%)	Not available	11 (8.1)	7 (9.3)	0 (0.0)	4 (10.5)
	Negative	11 (8.1)	6 (8.0)	3 (13.6)	2 (5.3)
	Positive	113 (83.8)	62 (82.7)	19 (86.4)	32 (84.2)
RF, *n* (%)	Negative	49 (36.3)	24 (32.0)	8 (36.4)	17 (44.7)
	Positive	86 (63.7)	51 (68.0)	14 (63.6)	21 (55.3)

Only anti-CCP2^+^ at-risk individuals who progressed to inflammatory arthritis and had one or more US scan prior to inflammatory arthritis development are included.

* Individuals in whom the most recent scan before progression was negative and where this scan was >6 months before progression.

US subclinical synovitis was detected in the following anatomical areas: wrists in 45/75 (60.0%) CCP2^+^ individuals, MCP joints in 30/75 (40.0%), MTP joints in 22/75 (29.3%), PIP joints in 17/75 (22.7%), knees in 6/75 (8.0%), elbows in 1/75 (1.3%) and ankles in 1/75 (1.3%).

### Predicting the development of US subclinical synovitis

A total of 220 CCP2^+^ individuals with a normal baseline US scan (i.e. no US synovitis or bone erosions) who had one or more longitudinal US scan and in whom a complete dataset was available were included in this analysis. The clinical and demographic characteristics of individuals included are reported in Table 2.

**Table 2:** Baseline demographic, clinical and imaging characteristics of the anti-CCP2^+^ at-risk individuals

Characteristics		Baseline (*N* = 220)	Longitudinal analyses			
			Developed US subclinical synovitis (ever) (*n* = 69)	Developed US subclinical synovitis (12 months) (*n* = 28)	Developed US subclinical synovitis (24 months) (*n* = 49)	Did not develop subclinical US synovitis (ever) (*n* = 151)
Age, years, mean (s.d.)		48.9 (12.4)	53.0 (10.8)	53.2 (9.6)	52.3 (10.9)	24.9 (12.6)
Female, *n* (%)		164 (74.5)	49 (71.0)	20 (71.4)	33 (67.3)	115 (76.2)
Tenderness in the hands, *n* (%)		71 (32.3)	20 (28.2)	8 (28.6)	12 (24.5)	51 (33.8)
EMS, minutes, median (IQR)		5 (0–30)	10 (0–30)	30 (2.5–60)	20 (0–60)	0 (0–30)
Anti-CCP2 antibodies, *n* (%)	Low	83 (37.7)	19 (27.5)	8 (28.6)	13 (26.5)	64 (42.4)
	High	137 (62.3)	50 (72.5)	20 (71.4)	36 (73.5)	87 (57.6)
Anti-CCP3 antibodies, *n* (%)	Negative	109 (49.5)	17 (24.6)	7 (25.0)	11 (22.4)	59 (39.1)
	Positive	111 (50.5)	52 (75.4)	21 (75.0)	38 (77.6)	92 (60.9)
RF, *n* (%)	Negative	161 (73.2)	39 (56.5)	16 (57.1)	28 (57.1)	122 (80.8)
	Positive	59 (26.8)	30 (43.5)	12 (42.9)	21 (42.9)	29 (19.2)

Only anti-CCP2^+^ at-risk individuals with normal baseline US scan who had one or more longitudinal US scan and in whom a complete dataset was available are included.

In 220 CCP2^+^ at-risk individuals with normal baseline US scans who had one or more longitudinal US scan and a complete dataset, US synovitis was detected in 69/220 (31.4%) [median time to first developing US synovitis: 56.4 weeks (IQR 33.0–112.0); median number of US scans: 2.0 (IQR 1.0–3.0); median number of joints with US synovitis: 2.0 (IQR 1.0–2.0)].

US subclinical synovitis was detected on longitudinal scans in the following anatomical areas: wrists in 44/69 (63.8%) CCP2^+^ individuals, MCP joints in 30/69 (43.5%), MTP joints in 14/69 (20.3%), knees in 11/69 (15.9%), PIP joints in 4/69 (5.8%) and elbows in 1/69 (1.4%).

In the univariable analysis, age [OR 1.04 (95% CI 1.02, 1.07), *P* < 0.01], high level anti-CCP2^+^ [OR 1.94 (95% CI 1.04, 3.60), *P* = 0.04], anti-CCP3 antibodies [OR 4.77 (95% CI 2.52, 9.03), *P* < 0.01] and RF [OR 3.24 (95% CI 1.73, 6.05), *P* < 0.01] were predictive for the development of US subclinical synovitis on subsequent US scans. In the multivariable analysis, only anti-CCP3 antibodies remained significantly predictive [OR 4.75 (95% CI 1.97, 11.46), *P* < 0.01] while borderline results were observed with age [OR 1.04 (95% CI 1.01, 1.07), *P* = 0.01] (Table 3). The predictive value of anti-CCP3 for US subclinical synovitis development was also observed when the multivariable analysis was carried out excluding high-level anti-CCP2 [anti-CCP3 OR 3.60 (95% CI 1.74, 7.44), *P* < 0.01] or RF [anti-CCP3 OR 5.64 (95% CI 2.50, 12.76), *P* < 0.01] (Supplementary Table S1, available at *Rheumatology* online). Interestingly, RF was predictive for the development of US subclinical synovitis when anti-CCP3 antibodies were not included in the multivariable analysis [RF OR 2.61 (95% CI 1.34, 5.08), *P* = 0.01]. No significant collinearity between anti-CCP2, anti-CCP3 and RF was found (V = 0.54, 0.28 and 0.52 for anti-CCP2/anti-CCP3, anti-CCP2/RF and anti-CCP3/RF, respectively).

**Table 3:** Univariable and multivariable regression analyses for the development of US synovitis

Variables	Univariable analysis		Multivariable analysis	
	OR (95% CI)	*P*-value	OR (95% CI)	*P*-value
Gender (male)	1.30 (0.69, 2.48)	0.42	–	–
Age	**1.04 (1.02, 1.07)**	**<0.01**	**1.04 (1.01, 1.07)**	**0.01**
Tenderness in the hands	1.25 (0.67, 2.32)	0.48	–	–
EMS	1.00 (0.99, 1.01)	0.11	–	–
Anti-CCP2^+^ (high level)	**1.94 (1.04, 3.60)**	**0.04**	0.60 (0.26, 1.41)	0.24
Anti-CCP3^+^	**4.77 (2.52, 9.03)**	**<0.01**	**4.75 (1.97, 11.46)**	**<0.01**
RF^+^	**3.24 (1.73, 6.05)**	**<0.01**	1.46 (0.70, 3.05)	0.31

Significant results are in bold.

**Fig. 2:**
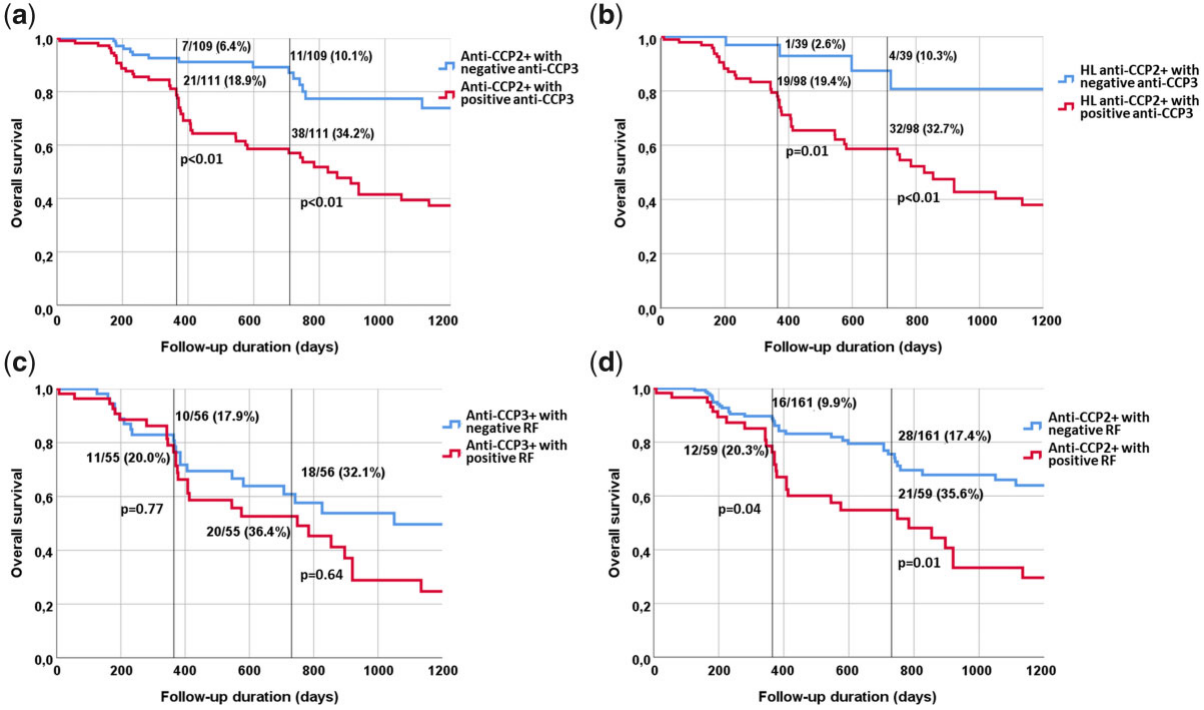
Kaplan–Meier analysis shows US subclinical synovitis-free survival time in CCP2^+^ at-risk individuals Percentages refer to the individuals who developed US subclinical synovitis at 12 and 24 months follow-up (black lines). HL: high level.

CCP2^+^ individuals with positive anti-CCP3 antibodies show a significantly reduced US subclinical synovitis-free survival rate compared with individuals with negative anti-CCP3 antibodies (Fig. 2). At the 1 and 2 year follow-up, 18.9% and 34.2%, respectively, of individuals with dual CCP2/CCP3 positivity developed subclinical synovitis on longitudinal scans compared with 6.4% and 10.1% of CCP2^+^ individuals with negative anti-CCP3 antibodies (*P* < 0.01 for both) (Fig. 2a). Similar results were observed in the subgroup of high-level CCP2^+^ individuals at the 1 and 2 year follow-up; respectively, 19.4% and 32.7% of high-level CCP2^+^/anti-CCP3^+^, but only 2.6% and 10.3% of high-level CCP2^+^ individuals with negative anti-CCP3 antibodies developed subclinical synovitis on longitudinal scans (*P* = 0.01 and *P* < 0.01, respectively) (Fig. 2b). The impact of RF on subclinical synovitis-free survival rate was not significant in individuals with positive anti-CCP3 antibodies (Fig. 2c). Conversely, this was notable in individuals with anti-CCP2^+^/RF^+^ [i.e. 21/59 (35.6%) developed US subclinical synovitis within 2 years of follow-up] compared with individuals with positive anti-CCP2^+^ and negative RF [i.e. 28/161 (17.4%), *P* = 0.01] (Fig. 2d).

## Discussion

**Fig. 3:**
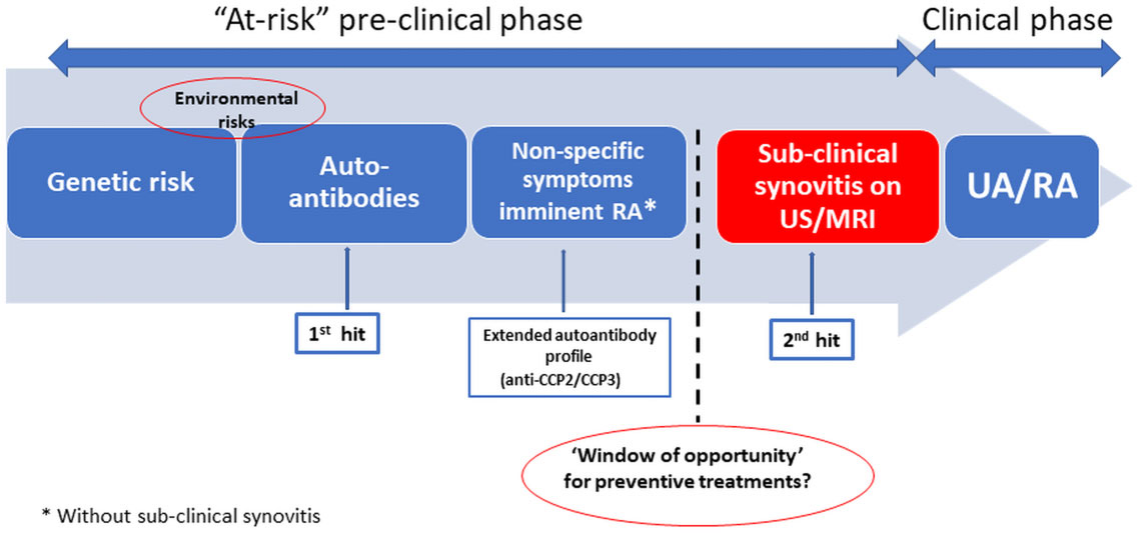
The ‘disease continuum' of CCP2^+^ at-risk individuals Anti-CCP^+^ at-risk individuals with MSK symptoms but without any joint involvement (clinical or subclinical) may represent the critical time point for timing interventions to prevent the onset of joint disease. UA: undifferentiated arthritis.

The results of the current study show that in CCP2^+^ at-risk individuals with MSK symptoms, but without clinical synovitis, the majority of progressors go through a stage of US subclinical joint inflammation prior to developing inflammatory arthritis; this represents a distinct stage of the ‘RA continuum’. Moreover, this is an important group to recognize, as it also represents the first transition of systemic autoimmunity into articular inflammation, the so-called ‘second hit' in the pathogenesis of RA [24] (Fig. 3).

There is increasing evidence from patient-reported outcomes and the very high predictive value of subclinical synovitis on imaging that the presence of subclinical synovitis in multiple joints may represent a distinct disease in its own right [25, 26]. In support of this, serial US assessments in a cohort of anti-CCP2^+^ at-risk individuals suggest that subclinical synovitis occurs directly before the development of clinical synovitis [27] and the fact that the majority of anti-CCP2^+^ individuals with US synovitis who develop inflammatory arthritis do so within 12 months of follow-up [15, 16]. Of note, in our study, >60% of progressors had evidence of subclinical synovitis on US scans performed within 6 months prior to inflammatory arthritis development.

Certainly one might argue that US subclinical inflammation represents a late feature in the development of inflammatory arthritis and, when present, may represent an inevitable progression to clinical synovitis, especially when multiple joints are affected; patients who have progressed this far already have joint inflammation and therapy may only delay eventual disease progression rather than truly preventing arthritis [28]. Instead, anti-CCP^+^ individuals with MSK symptoms in whom subclinical joint inflammation is not present but imminent may represent ‘ideal targets’ for therapeutic trials for arthritis prevention. Halting disease progression in this population would prevent the development of any joint inflammation, the cardinal feature of RA, and would likely produce better outcomes. This is also a valuable population to use for investigating the immunopathology of RA development, as the immunological events that drive the initial joint involvement in RA (i.e. the ‘second hit') are not clear. The position of this population along the early ‘RA continuum' is well suited to address this.

The results of the present study show that anti-CCP3 antibodies have a predictive role for the imminent development of subclinical synovitis in CCP2^+^ at-risk individuals. Such predictive value was not replicated with either high-level anti-CCP2 antibodies or RF, neither of which were predictive for the development of US synovitis in the multivariable analysis (Table 3). However, it should be noted that RF was predictive for the development of US subclinical synovitis when anti-CCP3 antibodies were not included in the multivariable analysis. Of note, around a third of high-level anti-CCP2^+^/CCP3^+^ individuals developed US subclinical synovitis within 2 years of follow-up. On the other hand, only 4/39 (10.3%) high-level anti-CCP2^+^/CCP3^−^ individuals developed US subclinical synovitis on one or more longitudinal scan in this time frame (Fig. 2b). Thus the CCP3 result had a 3-fold effect on the prognostic value of high-level anti-CCP2 antibodies. As shown in Fig. 2d, a similar trend was also observed in individuals with double positive anti-CCP2 and RF in comparison with individuals with positive anti-CCP2 but negative RF.

Our group has previously demonstrated the predictive role of anti-CCP3 for the development of inflammatory arthritis [29]. This is likely to be related to dual anti-CCP2/CCP3 positivity reflecting an expanded ACPA repertoire (i.e. different antigenic targets/ACPA fine specificity being detected) and suggests that in anti-CCP2^+^ at-risk individuals, anti-CCP3 antibodies may be identifying a more advanced stage of autoimmunity driving the onset of subclinical and later clinical inflammation [29, 30].

However, progression to inflammatory arthritis in at-risk individuals with RA-related antibodies and MSK symptoms is not always inevitable, even in those with US subclinical joint inflammation. In a recent study, baseline US synovitis was not associated with the development of clinical arthritis at 12 months follow-up in 54% of ACPA^+^ individuals with arthralgia [31]. The rate of progression to inflammatory arthritis in at-risk individuals with subclinical synovitis is variable and depends on several factors, such as the target population (i.e. asymptomatic first-degree relatives *vs* individuals with MSK symptoms and RA-related antibodies), distribution and number of joints or tendons involved and the type of US pathological findings detected (e.g. grey-scale synovitis, PD signal) [32]. In the current study, progressors were found to have a median of 2 joints with US subclinical synovitis (IQR 1.0–3.0) prior to inflammatory arthritis development. US subclinical synovitis was mainly detected in the wrists, MCP joints and MTP joints (60.0%, 40.0% and 29.3% of progressors, respectively). Indeed, further research is needed to establish which joints, and indeed how many joints, need to be imaged for optimum predictive accuracy. Most of the US studies in at-risk cohorts have adopted comprehensive US protocols that evaluate multiple pathological findings (i.e. PD signal, grey-scale synovitis and/or bone erosions), including most or all relevant small joints. In addition, the interpretation of US-detected synovitis should be performed in the context of other joint findings. Indeed, it is well known that inflammation accompanies structural changes of OA in small and large joints [33].

Our results suggest that anti-CCP2^+^/CCP3^+^ individuals without subclinical joint disease are at a critical transition point in the evolution of RA (i.e. the transition from autoimmunity to joint inflammation). This transition to the first detectable phase of joint involvement may represent the so-called ‘second hit' in RA pathogenesis and may be viewed as an additional distinct stage in the ‘RA continuum'. Moreover, this point may represent a unique time point for preventing the onset of joint disease. However, caution is warranted not to dismiss the possibility of participation in prevention trials of those who already have subclinical joint inflammation.

To our knowledge, this is the first study aimed at identifying at-risk individuals just before the development of subclinical joint involvement. Indeed, this novelty is the main strength of the current study. Moreover, data are presented from one of the largest prospective cohorts with the longest follow-up of CCP^+^ at-risk individuals. However, all individuals were anti-CCP2^+^. Therefore the results of our study can be interpreted only in the context of anti-CCP2^+^ at-risk individuals.

## Conclusions

In CCP2^+^ at-risk individuals with MSK symptoms but without clinical synovitis, the majority of progressors go through a stage of US subclinical joint inflammation prior to the development of inflammatory arthritis, thus representing a distinct and important stage of the ‘RA continuum'.

Anti-CCP3 antibodies have a potential role in the identification of CCP2^+^ individuals who are about to develop clinical or subclinical RA-related joint inflammation (i.e. before the ‘second hit' in RA pathogenesis occurs). This may be the ideal population for interventions to prevent joint disease. This is also a unique population for investigating the drivers of joint involvement in the development of RA.

## Supplementary Material

keab862_Supplementary_Data

## Data Availability

Data are available upon reasonable request.
