# Supplementary material for: Ultrasound subclinical synovitis in anti-CCP-positive at-risk individuals with musculoskeletal symptoms: an important and predictable stage in the rheumatoid arthritis continuum
Source: Rheumatology (Oxford). 2021 Nov 24;61(8):3192–200. doi: 10.1093/rheumatology/keab862 (PMC9348771; doi:10.1093/rheumatology/keab862)
Supplement: keab862_Supplementary_Data [file keab862_supplementary_data.docx]

**Supplementary Table S1.** Multivariable regression models for the development of US synovitis excluding anti-CCP2+ (high level) or anti-CCP3+ or RF+ individuals.

|  | **Multivariable analysis** |  |
| --- | --- | --- |
|  | OR (95% CI) | p-value |
|  | **Model 1 (no anti-CCP2 high level+)** |  |
| Age | 1.03 (1.01-1.06) | 0.01 |
| RF+ | 1.47 (0.71-3.05) | 0.31 |
| CCP3+ | 3.60 (1.74-7.44) | <0.01 |
|  | **Model 2 (no anti-CCP3+)** |  |
| Age | 1.04 (1.01-1.06) | 0.01 |
| RF+ | 2.61 (1.34-5.08) | 0.01 |
| CCP2+ (high level) | 1.29 (0.66-2.53) | 0.45 |
|  | **Model 3 (no RF+)** |  |
| Age | 1.04 (1.01-1.07) | 0.01 |
| CCP3+ | 5.64 (2.50-12.76) | <0.01 |
| CCP2+ (high level) | 0.60 (0.26-1.40) | 0.24 |

**Legend**. **95%CI**: 95% of the confidence interval, **CCP2**: second generation anti-cyclic citrullinated peptide, **CCP3:** third generation anti-cyclic citrullinated peptide, **CI:** confidence interval**, RF**: rheumatoid factor, **US:** ultrasound.
